# Pangenomics to understand prophage dynamics in the Pectobacterium genus and the radiating lineages of Pectobacterium brasiliense

**DOI:** 10.1099/mgen.0.001392

**Published:** 2025-05-07

**Authors:** Lakhansing A. Pardeshi, Inge van Duivenbode, Michiel J.C. Pel, Eef M. Jonkheer, Anne Kupczok, Dick de Ridder, Sandra Smit, Theo A. J. van der Lee

**Affiliations:** 1 Bioinformatics Group, Wageningen University & Research, Droevendaalsesteeg 1, 6708PB, Wageningen, Netherlands; 2 Biointeractions and Plant Health, Wageningen Plant Research, Droevendaalsesteeg 1, 6708PB, Wageningen, Netherlands; 3 Dutch General Inspection Service for Agricultural Seeds and Seed Potatoes (NAK), Randweg 14, 8304 AS Emmeloord, Netherlands; 4 Netherlands Institute for Vectors, Invasive Plants and Plant Health (NIVIP), National Plant Protection Organization (NPPO), Netherlands Food and Consumer Product Safety Authority (NVWA), Geertjesweg 15, 6706 EA Wageningen, Netherlands

**Keywords:** pangenome, *Pectobacterium*, prophages

## Abstract

Bacterial pathogens of the genus *Pectobacterium* are responsible for soft-rot and blackleg diseases in a wide range of crops and have a global impact on food production. The emergence of new lineages and their competitive succession is frequently observed in *Pectobacterium* species, in particular in *Pectobacterium brasiliense*. With a focus on one such recently emerged *P. brasiliense* lineage in the Netherlands that causes blackleg in potatoes, we studied genome evolution in this genus using a reference-free graph-based pangenome approach. We clustered 1,977,865 proteins from 454 *Pectobacterium* spp*.* genomes into 30,156 homology groups. The *Pectobacterium* genus pangenome is open, and its growth is mainly contributed by the accessory genome. Bacteriophage genes were enriched in the accessory genome and contributed 16% of the pangenome. Blackleg-causing *P. brasiliense* isolates had increased genome size with high levels of prophage integration. To study the diversity and dynamics of these prophages across the pangenome, we developed an approach to trace prophages across genomes using pangenome homology group signatures. We identified lineage-specific as well as generalist bacteriophages infecting *Pectobacterium* species. Our results capture the ongoing dynamics of mobile genetic elements, even in the clonal lineages. The observed lineage-specific prophage dynamics provide mechanistic insights into *Pectobacterium* pangenome growth and contribution to the radiating lineages of *P. brasiliense*.

**Impact Statement**

The *Pectobacterium* genus is composed of diverse species that cause softrot in plants around the globe. Our genus-level pangenomic approach to understanding the periodic emergence of new lineages showed that the *Pectobacterium* pangenome continues to expand and integrated bacteriophages, i.e. prophages, are ubiquitously present in *Pectobacterium* genomes. The method developed to trace prophages across the pangenome captured not only prophages that are specific to a particular strain or species but also prophages that infect multiple species. This study demonstrates the necessity of pangenomic surveillance for efficient monitoring of plant pathogens and unravelling the dynamics of the mobilome in species evolution.

## Data Summary

All the scripts used for preprocessing and analysis are available in the GitHub repository, https://lakhanp1.github.io/Pectobacterium_pangenome. Notebooks with data analysis, figures and tables can be accessed at https://lakhanp1.github.io/Pectobacterium_pangenome/scripts/notebooks. Zenodo DOI for the GitHub repository: https://doi.org/10.5281/zenodo.12772014. Sequencing data can be accessed at National Center for Biotechnology Information (NCBI) with BioProjects PRJNA1122109 for the NIVIP collection and PRJNA1140644 for the NAK collection. Individual BioSample identifiers for all the sequenced genomes are listed in Table S10.

## Introduction

In plants, soft rot disease is mainly caused by bacteria from the *Pectobacterium* and *Dickeya* species, belonging to the *Pectobacteriaceae* family. *Pectobacterium* spp. is among the top ten plant pathogens and infects potatoes, ornamental plants and vegetable crops [1]. Although several of *Pectobacterium* species, such as *Pectobacterium brasiliense*, *Pectobacterium atrosepticum*, *Pectobacterium versatile*, *Pectobacterium parmentieri*, *Pectobacterium polaris* and *Pectobacterium punjabense*, cause soft-rot disease in plants [2], *P. brasiliense* is one of the dominant species, known to infect at least 19 different plant species across the world [3]. *P. brasiliense* was first detected and characterized as a pathogen associated with blackleg (BL) disease of potato (*Solanum tuberosum* L.) in Brazil and initially named *Erwinia carotovora* subsp. *brasiliensis* [4]. Subsequent molecular, phylogenetic, *in silico* DNA–DNA hybridization and average nucleotide identity (ANI) analysis led to the elevation of this taxon to the species level [5,8].

*Pectobacterium* species are known to co-infect [9,11] and show all three inter-species interaction behaviours – competition, cooperation and commensalism [12]. Until 2013, *Dickeya* spp. and *P. atrosepticum* were dominant in Western European countries like the Netherlands and Switzerland. After the initial reports of *P. brasiliense* identification in Western Europe in 2013, its abundance increased rapidly, and it is now detected in almost 70–80% of the BL diseased plants [13,15]. Strain specificity towards infecting certain host plants [3] and the ability to cause latent infections [16] are characteristics of *P. brasiliense*. This species shows the regular emergence of new lineages, also evident from the observed genomic diversity and a broad range of intra-species ANI [17]. Recent pangenome studies on the *Pectobacterium* genus have shown a growing pangenome, indicating that not all diversity is captured in this genus yet [17,18].

In prokaryotes, various mechanisms are responsible for the evolution of such heterogeneous strains, such as mutations and horizontal gene transfer, for example, via plasmids and bacteriophages (commonly called phages). Genomes for many species belonging to the *Pectobacterium* genus were assembled at single contig level with minimal evidence of autonomous plasmids [19,21], and yet, computational analyses predicted the presence of plasmids in the other 89 accessions [22]. Some phages can integrate into a prokaryotic genome through the lysogenic pathway and are called prophages. A study on a small collection of 54 *Pectobacterium* spp. genomes showed the presence of prophage-like sequences [23]. Prophages can influence physiological functions and confer significant fitness advantages to their bacterial hosts. These benefits include superinfection exclusion, acquisition of virulence factors and antibiotic resistance genes [24,28], also noted in *Pectobacterium* pathogens [29]. From a wider perspective of population expansion, prophages are known to contribute to the competitive success and dispersal of a prokaryotic strain from the environmental reservoir [30,31]. Therefore, it is critical to understand the role of prophages in species and strain diversity and its contribution to population structure, evolution and epidemiology.

Two distinct groups of *P. brasiliense* strains were detected during annual surveys from 2016 to 2018 in the Netherlands: a clonal lineage with a high ability to cause BL and another, relatively diverse group unable to cause BL in potatoes in field trials [17]. A subsequent comparative pangenomic analysis enabled the design of new functional assays to detect these BL-causing isolates [32]. However, a new group of BL-causing isolates that escaped these assays was detected during ongoing annual surveys. A common observation is the emergence of new lineages of *P. brasiliense* in the Netherlands, followed by their dominance over the existing lineages. We, therefore, extended the *Pectobacterium* genus pangenome by combining a genome collection from local epidemiological surveillance with global data to understand the mechanism of appearance and succession of the *P. brasiliense* strains. Further, we investigated the contribution of mobile genetic elements, and prophages in particular, in the growing *Pectobacterium* genus pangenome. Our results showcase an application of the pangenome approach to trace such mobile genetic elements, delineate their dynamics and provide mechanistic insights into pangenome growth.

## Methods

### Sample collection and phenotyping

During the annual screening and field inspection conducted by the Dutch General Inspection Service for Agricultural Seeds and Seed Potatoes (NAK) in the Netherlands from 2018 to 2020, *Pectobacterium* spp. strains were collected from symptomatic potato plants and non-symptomatic potato tubers. Non-symptomatic potato peels were crushed in water and incubated in a pectate buffer before spreading on single-layer crystal violet pectate (CVP) plates. Characteristic cavity-forming colonies were sub-cultured on the CVP and nutrient agar plates to obtain pure strains and stored at −80°C in 15% glycerol with half-strength nutrient broth.

The BL-causing ability of the *P. brasiliense* isolates was tested on potato cultivar Agria during 2019 to 2023 field trials. Isolates were grown on nutrient agar plates for 1 day at 28°C. Suspensions of OD_600_=0.1 were prepared in 10 mM phosphate buffer (pH 7.2) and diluted an extra 100×, resulting in a suspension of about 106 c.f.u. ml^−1^. Tubers were submerged in the solution and brought under a −0.07 Pa vacuum. The vacuum was kept for 10 min, after which tubers were left submerged for another 15 min. Tubers were air-dried before planting, and the ability to cause BL was scored as the development of typical plant symptoms [14]. Isolates that induced the BL phenotype in ≥30% of plants were categorized as BL-causing and in *<*5% plants as non-causing, and the remaining were labelled inconclusive. Additionally, 35 *Pectobacterium* species isolates were included from the Netherlands Institute for Vectors, Invasive Plants and Plant Health (NIVIP) collection.

### Genome sequencing, quality control and annotation

The 58 isolates in this collection are part of ongoing annual potato field inspections. Whole-genome sequencing and genome assembly were performed for 23 *P*. *brasiliense* isolates from the NAK collection and 35 isolates from the NIVIP collection as described in Jonkheer *et al*. [17] and Blom *et al*. [33], respectively. Publicly available genomes for *Pectobacterium* species until May 2022 and related BioSample metadata were downloaded using the National Center for Biotechnology Information (NCBI) E-utility tool [34]. For uniform taxonomy validation of our collection, we combined in-house and NCBI type strains to perform an independent ANI comparison and taxonomy correction. Of the 508 genomes, 26 marked as ‘anomalous’, ‘excluded’ or ‘replaced’ by NCBI were excluded. We used BUSCO (v5.2.2, database: ‘enterobacterales_odb10’) protein to evaluate genome assembly completeness and removed 21 genomes that had less than 99% ‘complete BUSCOs’ [35]. Finally, seven genomes were found to be duplicated, and hence, only one copy was kept for further analysis. To reduce potential artefacts caused by different gene prediction tools (or different versions of the same tool) used in NCBI genome assemblies, we reannotated the remaining 454 genomes using Prokka (v1.14.6) [36]. InterProScan (v5.56–89.0) was used to annotate genomes with protein family and domain information from the InterPro database [37,38]. EggNOG-mapper (v2.1.10) was used to annotate proteins by transferring annotations from the EggNOG database (v5.0.2) [39,40].

### Pangenome construction and analysis

A *Pectobacterium* genus pangenome was built using PanTools (v4.1.1) [41,42], which stores a hierarchical data structure containing a sequence-level De Bruijn graph with connected gene annotations and homology relationships among them. Functional annotations from InterProScan and EggNOG were added to the pangenome database using the *add_annotations* command. Homology grouping in PanTools employs mcl to cluster proteins with a similarity above a given threshold into groups [43]. The optimal settings for clustering are determined using the *optimal_grouping_*command [42], which evaluates eight different clusterings based on the organization of universal single-copy orthologs (BUSCO genes). Since this operation is computationally prohibitive on 454 genomes, we used 2 subsampling approaches. First, genomes of the type strain for each species were used. In the second, the *k*-mer tree built using the *kmer_classification* command was cut into 20 clusters (number of *Pectobacterium* species), and a genome was selected from each cluster at random. This second method of random subsampling was repeated 100× to generate 100 subsets of *Pectobacterium* genomes. The optimal grouping step of PanTools was performed on these subsets, and the relaxation setting with the highest average *F*-score was selected. This resulted in a relaxed setting of four, which corresponds to a minimum sequence similarity of 65%, an intersection rate of 0.05 for the *k*-mer set comparison, an inflation rate of 7.2 for Markov clustering and a contrast factor of five.

### Functional enrichment analysis

The genes with unknown function were assigned root Gene Ontology (GO) terms GO:0008150, GO:0003674 and GO:0005575 for biological process, molecular function and cellular component, respectively, by the annotation programs Prokka and InterProScan. The GO terms assigned to genes in PanTools’ neo4j database, including any root GO term, were consolidated at the homology group level. The GO graph topology-aware enrichment method [44] implemented in R (v4.2.1) BioConductor (v3.16) package and topGO (v2.50.0) was used to perform GO enrichment at the homology group level.

### Phylogenetic analysis

Core genes were used to construct a maximum likelihood phylogenetic tree using the PanTools *core_phylogeny* command [45]. The core-SNP tree was rooted using *Pectobacterium cacticida* as an outgroup. Phylogenetic trees were processed using R (v4.2.1) packages Ape (v5.7.1) [46,47] and treeio (v1.22.0) [48], and data were visualized with R packages ggtree (v3.6.2) [49] and ComplexHeatmap (v2.15.1) [50].

### Prophage detection and quality control

Prophages and plasmids were detected in genomes using the geNomad (v1.5.0) pipeline [51] on genome FASTA files. The geNomad tool uses a hybrid approach for mobile genetic element identification by combining neural network-based and marker gene-based classifiers on the demarcated genomic regions enriched with marker genes. Virus genome completeness assessment was evaluated using CheckV (v1.0.1) [52]. Host sequence regions at the prophage boundaries predicted by CheckV were trimmed to remove the contamination resulting from host genes. One hundred thirty-nine prophages without any gene of viral origin were excluded from downstream analysis. In fragmented genome assemblies, prophages may be split over multiple contigs, leading to an overestimation of the number of prophages. We used homology group information of the orthologous prophages from the high-quality genome assemblies to identify such fragmented prophages (*n*=145) and merged them into 61 prophages. Merging was considered confident if the merged prophages together covered at least 80% of the closest matching orthologous prophages (*n*=50 from 110 fragmented prophages), and the rest were excluded (*n*=11). Finally, the shortest prophage detected with *>*90% completeness was of length 5.2 kb, and hence, prophages smaller than 5 kb were also excluded, resulting in 1,369 prophages ≥5 kb in length.

A comparison of clonal isolate genomes (ANI *>*99.9%) from the TP-Pbr clade (*n*=35) showed predicted plasmids in fragmented genome assemblies (*n*=26) but not in the single chromosome assemblies (*n*=9). Furthermore, the plasmid homology groups from the isolates with fragmented genome assemblies could be mapped in tandem onto the chromosome-level assembly, suggesting a false-positive identification. Alternatively, these may be integrated plasmids in the single chromosome genomes, also known as episomes; this is supported by the consistent observation of contigs of length 293 kb and 89 kb as putative plasmids in the fragmented genome assemblies. Nevertheless, this renders the plasmid prediction results unreliable, and hence, they were not considered further in the current study.

### Prophage gene function categorization

PFAM and COG annotations were manually categorized into relevant Prokaryotic Virus Remote Homologous Groups database (PHROGs) categories using a keyword-based approach (Table S8, available in the online Supplementary Material). Anti-phage defence systems were initially identified using DefenseFinder (v1.2.0) [53] (Table S9). Subsequently, the keyword-based strategy was also employed to identify additional defence system genes that were missed by DefenseFinder.

### Orthologous prophage detection

Each putative prophage region was represented as a sequence of homology groups from the pangenome, defined as the prophage signature. Prophage similarity was quantified by evaluating the syntenic conservation of homology groups between two prophage signatures. To quantify such syntenic conservation, we applied dynamic programming to align two prophage signatures and calculate the pairwise Jaccard index. Only syntenically matching homology groups between a pair of prophage signatures are considered for determining the size of the intersection for the Jaccard index calculation. During dynamic programming scoring, a score of 5 was used for a match and −2 for a mismatch of homology groups between two prophage signatures being compared (Fig. S9). To account for the natural variation in prophages due to mosaicism and evolution, a maximum gap length of two was allowed. A syntenic Jaccard index was considered significant if two prophages had the longest common subsequence with a chain length, i.e. the number of syntenicly matching homology groups, of at least five. An all-vs-all syntenic Jaccard index matrix was clustered using the complete linkage hierarchical clustering function ‘hclust’ in R. A conserved phage tail-like bacteriocin, carotovoricin (CTV), was present in 428/454 *Pectobacterium* genomes and clustered together. We used CTV’s intra-cluster similarity as a threshold to decide the grouping of similar prophage-like elements into clusters. The complete-linkage dendrogram was cut at a height 0.66, which was just above the node height for the CTV cluster, resulting in 436 prophage clusters. Here, in addition to being simple and easily interpretable, the complete-linkage clustering ensures that each resulting cluster includes prophages with intra-cluster similarity equal to or higher than the chosen cut-height or similarity threshold. A cluster representative backbone was selected using criteria in the following order: highest CheckV completeness score, longest prophage length and highest average intra-cluster Jaccard index.

### Prophage cluster visualization

A 5 kb flanking region for each prophage was included to provide a broader context to understand integration at the same or different sites in the genome. An R script was written to extract the homology groups for prophages in a cluster, and the data were stored in the required JSON format by clustermap.js (https://github.com/gamcil/clustermap.js). This JSON data was used in clinker [54] to generate interactive plots to visualize orthologous prophages. Prophage genes were colored as per the PHROGs categories described above.

## Results

### Emergence of BL-causing *P. brasiliense* isolates that evade diagnostics

**Fig. 1.**
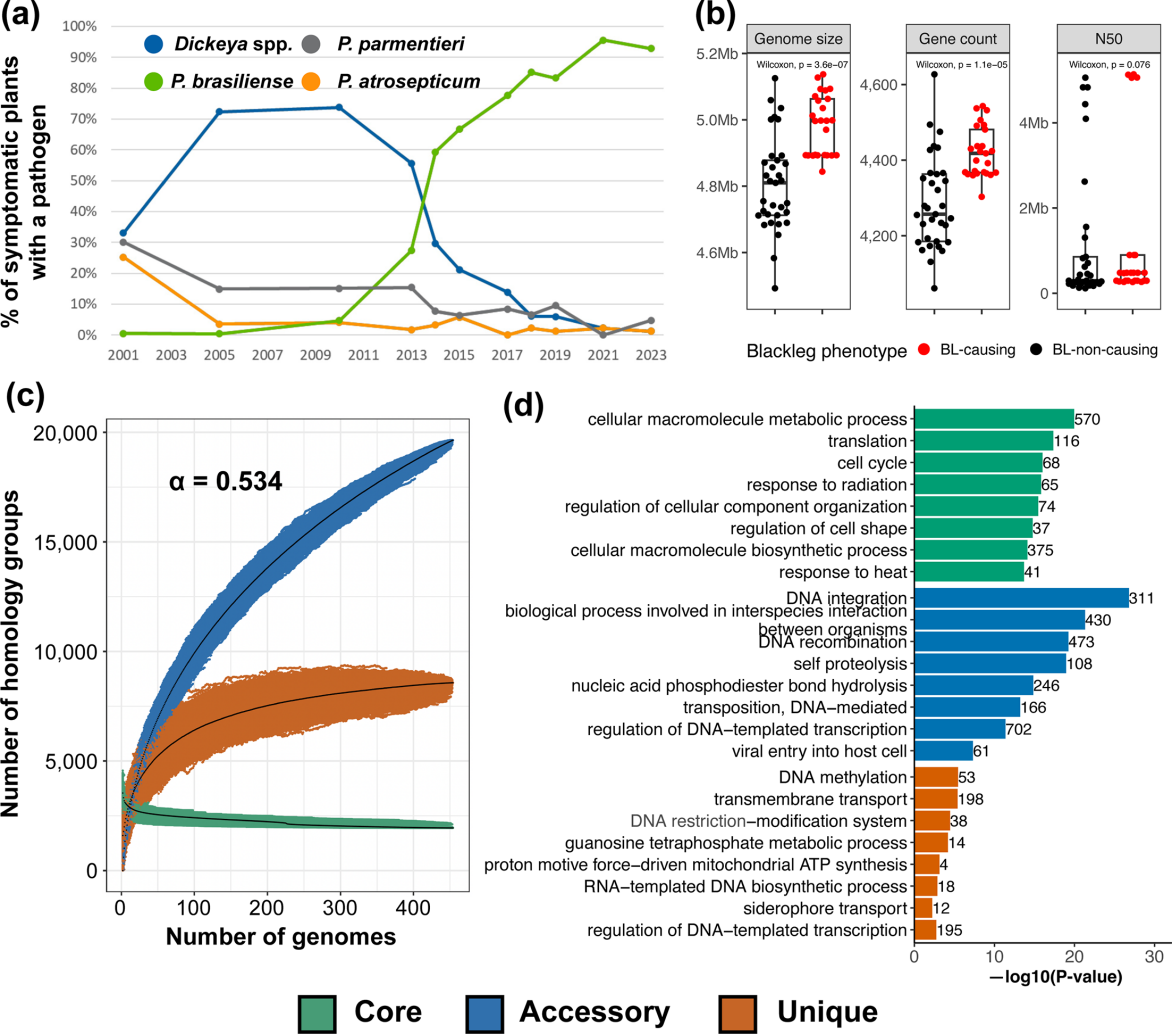
The *Pectobacterium* genus pangenome summary. (**a**) Percentage of different pathogens identified from the plants showing BL symptoms during the annual inspection in the Netherlands. (**b**) In the Dutch collection, BL-causing isolates have larger genomes and more protein-coding genes than BL-non-causing isolates. (**c**) Pangenome growth curves were simulated by randomly sampling 10,000 sets of genomes of a specific size. For each genome set, the number of core, accessory and unique genes are plotted as points in different colours. The black lines trace the medians of respective homology group categories. The *Pectobacterium* pangenome has a stable core but an increasing accessory genome. Heaps’ law *alpha*=0.534 is *<* 1 and implies an open pangenome. (**d**) Functional enrichment of GO biological processes in the core, accessory and unique pangenome.

Yearly inspection of potato fields, coupled with PCR-based diagnosis, indicates that after 2013, *P*. *brasiliense* has emerged as the dominant pathogen in the Netherlands, replacing *Dickeya* spp. In recent years, we have observed a higher prevalence of *P. brasiliense* on BL and soft-rot infected plants, wherein 95% of symptomatic plant material shows infection by *P. brasiliense* (Fig. 1a). From 2018 to 2020, *P. brasiliense* isolates were sampled from symptomatic and asymptomatic potato (*S. tuberosum*) plants in the Netherlands. These isolates were genotyped as BL-causing or BL-non-causing based on diagnostic quantitative PCR assays [32]. Subsequent field trials showed that some of the presumed non-causing *P. brasiliense* isolates did induce the BL phenotype, implying a false negative PCR diagnosis. These isolates are henceforth referred to as ‘FN-Pbr’. The BL-causing *P. brasiliense* that are also positive for BL PCR diagnosis are referred to as ‘TP-Pbr’.

We also detected multiple *Pectobacterium* species on the same infected plant material during the inspection in the Netherlands. To assess the relation between the newly identified FN-Pbr isolates and the existing BL-causing isolates and to understand potential inter-species interactions, we sequenced 58 *Pectobacterium* spp*.* isolates collected in the Netherlands. This collection included 28 *P*. *brasiliense* isolates, 10 of which were FN-Pbr. Genome assemblies from public databases were leveraged to study FN-Pbr evolution at high resolution and further capture the genomic diversity in the *Pectobacterium* genus. At the time of analysis, the NCBI database contained 450 *Pectobacterium* spp. genomes. We added 396 high-quality genomes of these after curation (see ‘Methods’), resulting in a collection of 454 genomes representing 22 *Pectobacterium* species (Table S1, Figs S1 and S2).

### The growing *Pectobacterium* genus pangenome

To study the overall sequence diversity in our collection of genomes, we first performed pairwise ANI comparisons. Within the *P. brasiliense* clade, we observed a broad range of intra-species ANI scores: 9.8% of *P. brasiliense* genome pairs had ANI scores of 96% or less, and 1.8% of pairs had scores of 95% or less. Additionally, a genus-level ANI comparison showed at least five distinct *Pectobacterium* species for which inter-species ANIs were closer to 95%, a commonly used species delineation cutoff [55,56] (Fig. S3). For example, a clade with *P. versatile*, *Pectobacterium carotovorum* and *Pectobacterium odoriferum* and another clade with *P. polaris* and *Pectobacterium parvum*. Together, these results demonstrate a high genetic variability in *Pectobacterium* species, in particular for *P. brasiliense* in the Netherlands. Additionally, BL-causing *P. brasiliense* isolates had significantly larger genomes (Wilcoxon test, *P*=3.6×10^−7^) and more protein-coding genes (*P*=1.1×10^−5^) than BL-non-causing isolates (Fig. 1b). This was not due to differences in assembly quality, as there was no significant difference (*P*=0.076) in median assembly N50 values between the two groups and all genomes had BUSCO completeness ≥99%.

For a reference-free comparison of this heterogeneous genus, we constructed a genus-level pangenome of 454 *Pectobacterium* spp. genomes using PanTools [41]. In our pangenome, 1,977,865 protein-coding genes were clustered into 30,156 homology groups, which were further categorized into 1949 core (present in all genomes), 8,571 unique (exclusive to individual genomes) and 19,642 accessory groups. Fitting a Heaps’ law model to homology group counts as a function of newly added genomes provides an estimate of the pangenome openness, where a decay rate *α<*1 implies an open pangenome and *α>*1 a closed one [57]. In line with the earlier, smaller pangenomes [17,18], the *Pectobacterium* spp. pangenome is still open, with a decay rate *α* of 0.534 (Fig. 1c). In the species-level sub-pangenomes extracted from the genus-level pangenome, *P. versatile* and *P. brasiliense* are represented by significantly more genomes than others but surprisingly had the lowest decay rates of 0.501 and 0.538, respectively (Fig. S4, Table S2). After more than doubling the number of genomes in the genus-level pangenome compared to the previous version, the core remained stable with a small decrease of 83 homology groups (Fig. S5). The increase in the overall pangenome size by 7,815 homology groups was mainly attributed to the accessory category, with 6,474 additional homology groups (Table S2).

To gain broad functional insights on our pangenome, we performed a GO enrichment analysis on core, accessory and unique gene sets. The *Pectobacterium* core genome was enriched for elementary processes such as cell cycle, translation, heat response, macromolecule metabolism and ribosome assembly. The unique genome was enriched in DNA methylation, siderophore transport and lipid transmembrane transport. The accessory genome was enriched for DNA integration, recombination, transposition, viral entry into host, inter-species interaction etc. (Fig. 1d, Table S3).

### A new BL-causing *P. brasiliense* lineage

**Fig. 2.**
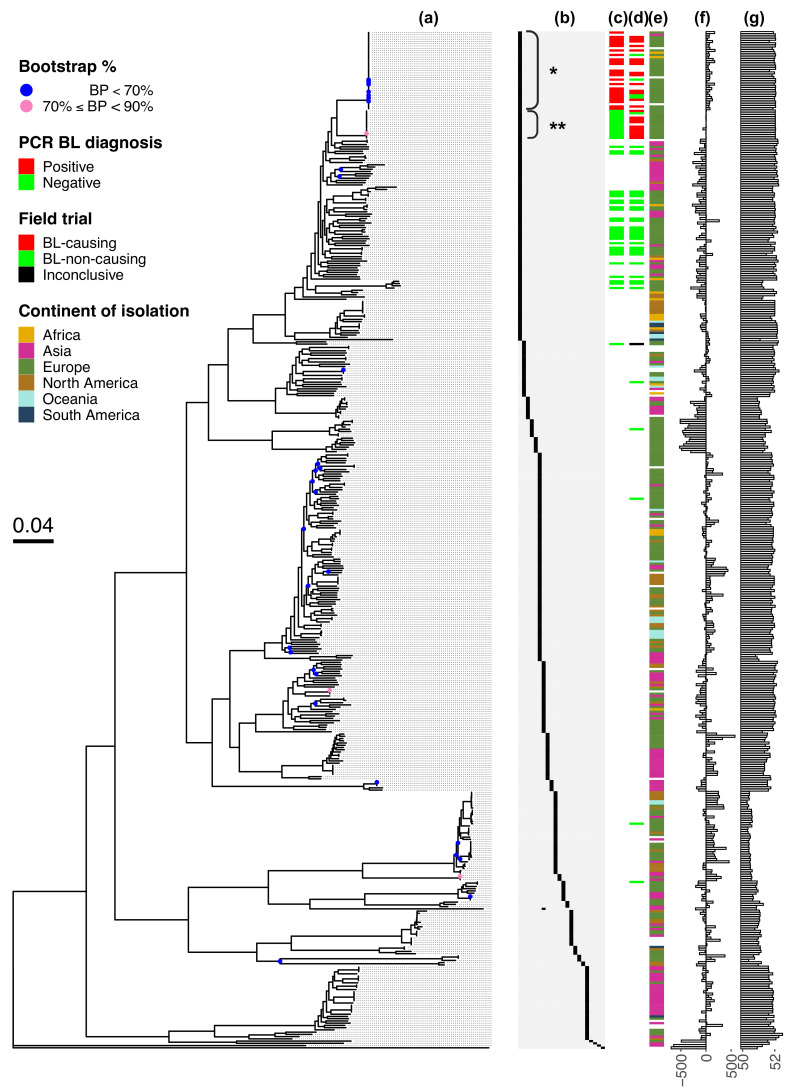
A new BL-causing *P. brasiliense* lineage. (**a**) A robust maximum likelihood tree was generated using 1,949 core gene alignments. Nodes with bootstrap values <90% and *<*70% are coloured pink and blue, respectively. The tree is rooted using *P. cacticida* as the outgroup. Metadata (from left to right): (**b**) a species key, where each column denotes a species ordered from left to right: *P. brasiliense*, *P. polaris*, *P. parvum*, *P. quasiaquaticum*, *P. aquaticum*, *P. versatile*, *P. carotovorum*, *P. odoriferum*, *Pectobacterium actinidiae*, *P. parmentieri*, *Pectobacterium wasabiae*, *P. punjabense*, *Pectobacterium polonicum*, *P. atrosepticum*, *Pectobacterium peruviense*, *Pectobacterium zantedeschiae*, *Pectobacterium betavasculorum*, *Pectobacterium aroidearum*, *Pectobacterium* sp. CFBP8739, *P. colocasium*, *Pectobacterium fontis* and *P. cacticida*. *: Clade enriched for TP-Pbr isolates. **: Clade enriched for FN-Pbr isolates. (**c**) PCR-based diagnosis of BL-causing ability of the isolates, (**d**) BL phenotype during field trials, (**e**) continent on which the isolate was collected, (**f**) difference in number of protein-coding genes concerning the median of 4,363 genes across the *Pectobacterium* genus and (**g**) GC% (lower limit of the bar plot shown is 50%).

A maximum likelihood phylogenetic tree based on the alignment of 1,949 core genes revealed that all 25 BL-causing *P. brasiliense* isolates were part of a monophyletic clade of 48 isolates (Fig. 2, Fig. S6). This BL-causing clade was split into two sub-clades: 1 of 35 isolates enriched for TP-Pbr and the other of 13 isolates enriched for FN-Pbr. Intra-clade pairwise comparison of the isolates within these sub-clades showed 100% ANI, implying almost identical genomes. The high level of ANI within BL-causing subclades (Fig. S3), along with their placement in core phylogeny (Fig. S6), suggests that FN-Pbr and TP-Pbr represent clonal lineages radiating from a BL-causing *P. brasiliense* common ancestor. Only one strain (NAK399) from the FN-Pbr sub-clade did not cause any BL phenotype in the field trials (0.5% infected plants). Combined, the chronology of identification and phylogenetic placement of all BL-causing *P. brasiliense* supports a pattern of periodic emergence of new lineages for this species.

### Contribution of prophage-like elements in the *Pectobacterium *pangenome

**Fig. 3.**
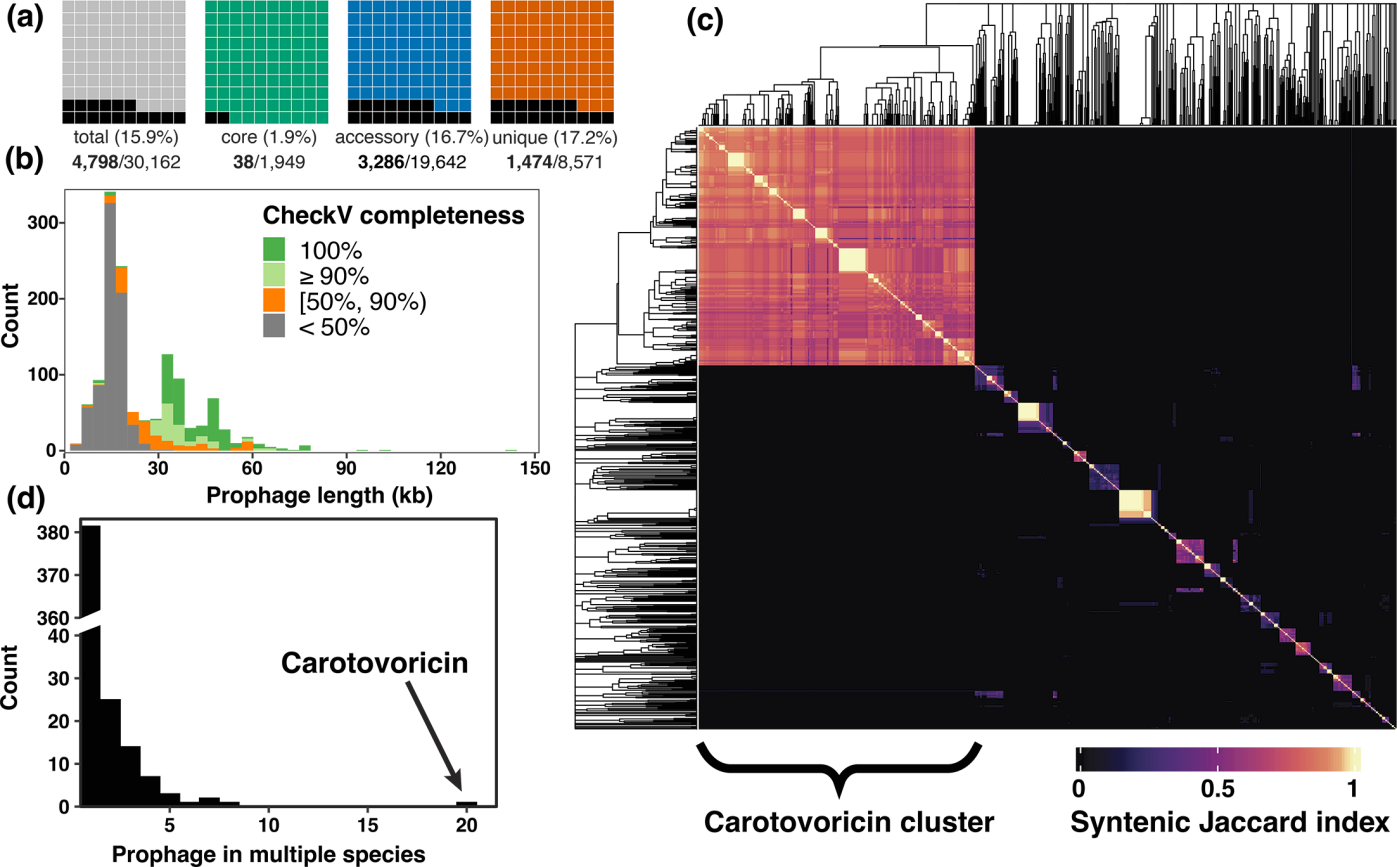
Prophage analysis. (**a**) Fraction of pangenome homology groups corresponding to prophages. (**b**) Prophage length distribution. (**c**) Clustering based on homology group content, represented by a heatmap of syntenic Jaccard index values. The largest cluster of CTV is annotated in the heatmap. The complete linkage dendrogram was cut at the node height of the CTV cluster to identify groups of orthologous prophages. (**d**) A histogram showing prophage backbone present in one or multiple *Pectobacterium* species. The CTV, a prophage-like element present in 20 species, is marked with an arrow.

Intrigued by our observation of the radiating lineages and heterogeneity in *P. brasiliense*, and enrichment of biological processes related to mobile genetic elements, we hypothesized such elements as an underlying factor in pangenome growth. Therefore, we systematically analyzed mobile genetic elements in *Pectobacterium* genomes using the geNomad pipeline [51] (see ‘Methods’ and Table S4 for details). A total of 1,369 prophage-like regions were identified in the 454 genomes, where all but one genome (g_177 of *P. brasiliense* strain NAK:433) contained at least one prophage (Table S5). Cumulatively, genes from prophages accounted for 4,801 (15.9%) of the pangenome homology groups. Among these prophage homology groups, 3,286 (16.7%) were accessory, 1,477 (17.2%) unique and only 38 belonged to the core genes in *Pectobacterium* genus pangenome (Fig. 3a, Table S6). Most of the prophage homology groups belonging to the core genes were located at the prophage borders, implying fuzzy prophage boundary estimation. The plasmid predictions made by geNomad were excluded because of inconsistencies between the complete and fragmented genomes of the clonal isolates (see ‘Methods’).

We used CheckV [52] to assess the completeness of the putative prophages and found 731 prophage-like elements with a completeness score of 50% or lower and 423 prophages with 90% or higher completeness (Fig. 3b, Table S4). The shortest prophage with 95% or more completeness was 5.4 kb long. The prophage length distribution shows three distinct peaks, with the highest peak at 16 kb mostly representing incomplete prophages, a peak at 35 kb representing prophages with completeness *>*90% and a peak at 50 kb comprising complete prophages. At the species level, *P. brasiliense* and *P. versatile* prophages contributed to ∼14% of their respective pangenomes (Table S6). This distribution is similar to the prophage fraction in the accessory genome of other highly dynamic bacterial pangenomes like *Escherichia coli* and *Salmonella enterica* [58]. Since the BL-causing *P. brasiliense* isolates have larger genomes, we compared the total prophage length between the two groups for a potential explanation. We observed that the BL-causing isolates had significantly larger median total prophage length (Wilcoxon test, *P*=6.9×10^−4^). This 50 kb difference observed in total prophage length accounts for 25% of the observed 200 kb disparity in median genome sizes between these two categories (Fig. S7).

The tailed bacteriophage class *Caudoviricetes*, with a double-stranded DNA genome, was represented most abundantly (*n*=1*,*357) in the *Pectobacterium* pangenome. Although much less abundant, other classes of viruses included filamentous phages of the *Inoviridae* (*n*=8) and *Microviridae* (*n*=2) families, a virus from *Patatavirales* order (*n*=1) and a 5.2 kb unclassified prophage (Fig. S8). *Inoviridae* and *Microviridae* are non-enveloped single-stranded DNA viruses, whereas *Patatavirales* is the largest order of non-enveloped positive-strand RNA viruses of plants and contains a single family *Potyviridae*, accounting for 30% of all known plant viruses [59]. While it was interesting to identify a plant virus of the order *Patatavirales* in the *P. parvum* genome assembly GCF_011378945.1, caution must be taken, as phages detected on independent contigs are difficult to ascertain as true integrations. Among the less abundant phages, host DNA integration evidence was observed for *Inoviridae* family prophages (Table S4); thus, these can be considered true prophages. The *Microviridae* and *Patatavirales* prophages were detected on an independent contig without flanking host genes and are likely the result of lytic viruses or sample contamination.

### Generalist prophages of *Pectobacterium* species

Bacteriophages have high genetic diversity and mosaicism is a hallmark of their genomes, primarily driven by horizontal gene transfer [60,63]. Given the variation in prophage length and the dominance of a single bacteriophage class, finding orthologous prophages would help in the characterization of prophage diversity and dynamics in the *Pectobacterium* pangenome. Prophages with similar gene content but different syntenies are likely the result of multiple recombination and mutation events and hence imply different evolutionary trajectories. Therefore, we used a conservative approach to identify orthologous prophages by calculating syntenic Jaccard indices of the prophage homology group signatures (Fig. S9). CTV, a conserved 16 kb phage tail-like bacteriocin occurring in 428 genomes, was used to decide the prophage grouping threshold (Fig. 3c) (see ‘Methods’). As a result, we identified 436 clusters of orthologous prophages (named phage_grp_1 to phage_grp436) and their respective cluster representative (Fig. S10, Table S7). These representative prophages covered a total of 4,493 homology groups (94% of the total 4,801), implying minimal loss of information during filtering and clustering.

**Fig. 4.**
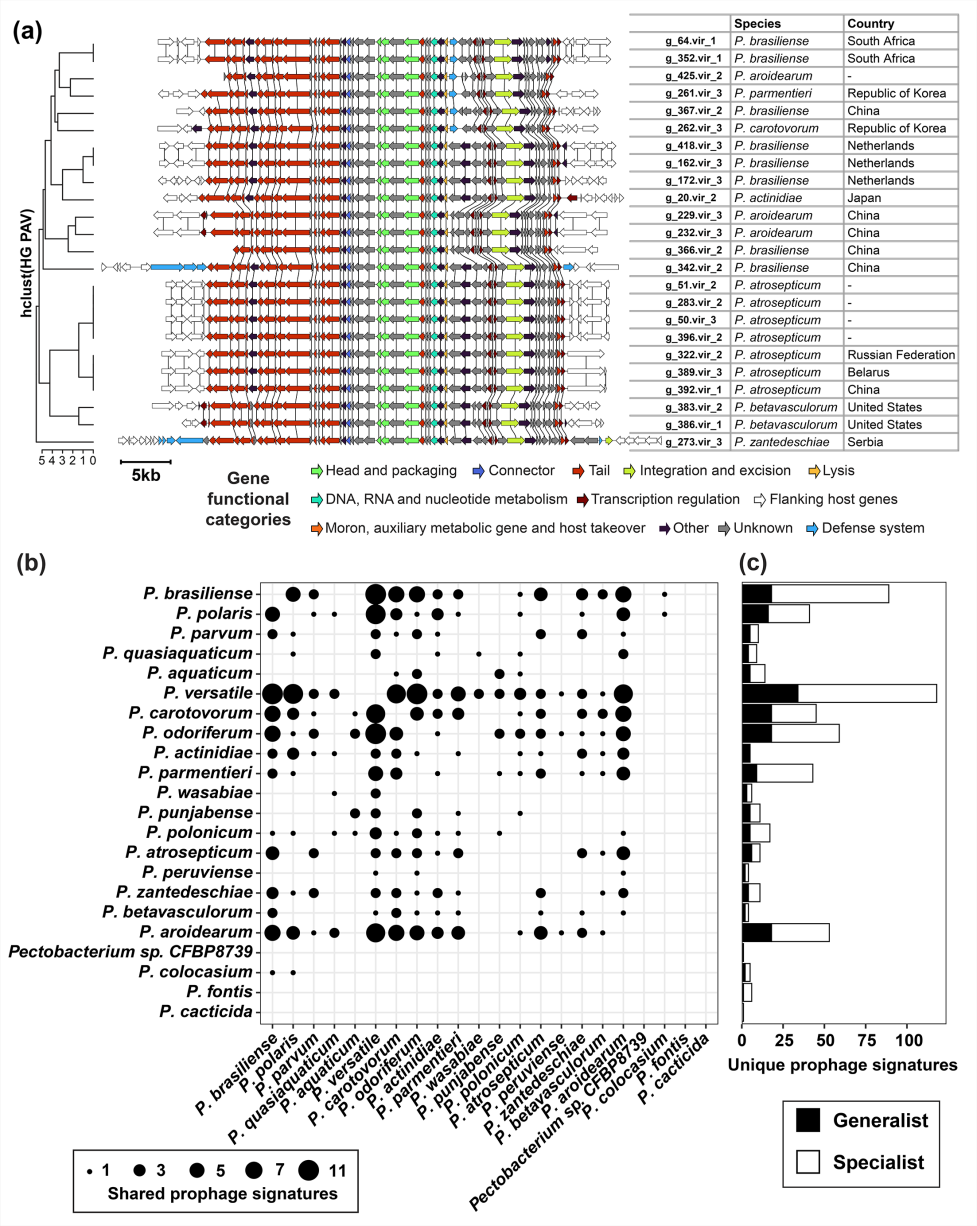
Generalist prophages infecting *Pectobacterium* species. (**a**) Prophage signatures of 22 members of the phage_grp_71 cluster. Flanking genes are coloured white, and the genes belonging to the same homology groups are connected with vertical lines. (**b**) Generalist prophages are shown as the overlap of prophage signatures between *Pectobacterium* species pairs. (**c**) Number of unique prophage signatures per species. The fraction of generalist prophages is indicated in black.

Most phages are species-specific and even strain/serovar/pathovar-specific [64,66], though broad-range phages targeting different species also exist [67,68]. To identify such generalist phages infecting multiple *Pectobacterium* species, we checked prophage clusters for the presence of multiple species. We identified 54 generalists and 382 species specialists(Fig. 3d). For example, clusters phage_grp_71 (Fig. 4a) and phage_grp_36 with 24 and 21 prophages, respectively, included complete prophages targeting multiple *Pectobacterium* species, and their hosts were collected from a wide spatiotemporal context (Table S7). No association was found between the *Pectobacterium* isolates harbouring phage_grp_71 phages and plant infection, as isolates were collected from both symptomatic and asymptomatic plants. Furthermore, none of the isolates harbouring phage_grp_36 phages were sampled from infected plants. *P. versatile* was the most frequent host of the generalist prophages, targeted by 34 out of 54 prophages (Fig. 4b,c) . The diverse ecological niches of *P. versatile* isolates, spanning water, soil and infected plants, likely contributed to their susceptibility to generalist phages. In contrast, the closely related species *Pectobacterium aquaticum* and *Pectobacterium quasiaquaticum* collected from freshwater bodies in France did not share any prophages yet did share prophage signatures with other *Pectobacterium* species.

### Prophage dynamics in the BL-causing clonal lineages

**Fig. 5.**
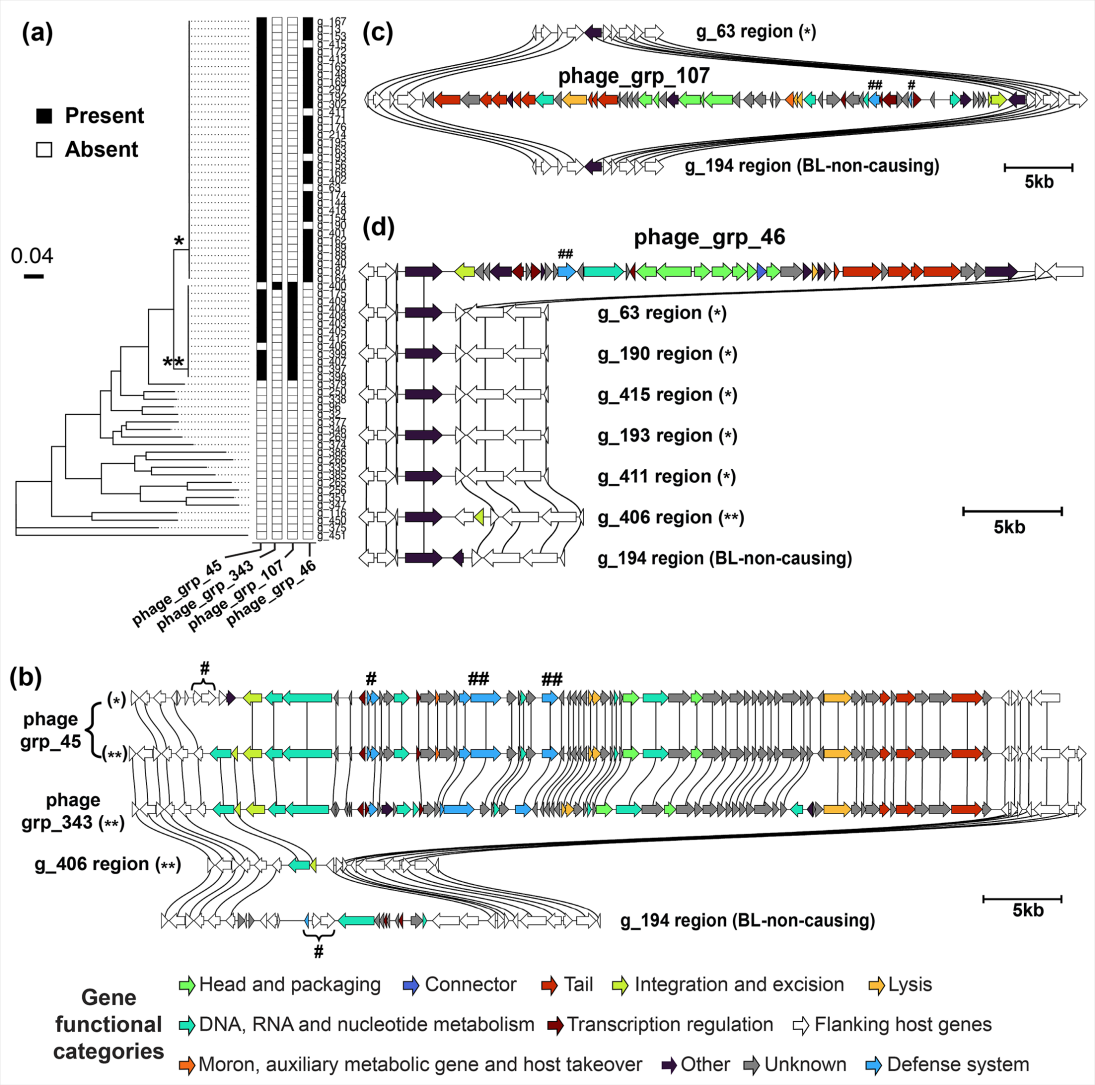
BL-causing *P. brasiliense* clade exclusive prophages. (**a**) Presence–absence (black-white) of the four prophages exclusive to BL-causing *P. brasiliense* lineage are shown next to a subtree for this clade. *: TP-Pbr clade, **: FN-Pbr clade. The remaining nodes in the tree belong to one representative *Pectobacterium* species. (**b**) BL-causing clade-specific prophage homology group signatures: five regions showing two representative prophages from the orthologous phage group phage grp 45, a single prophage from genome g 400 belonging to phage grp 343 group, a region from genome g 406 in which the prophage is absent and a region from BL-non-causing isolate genome g 194. (**c**) FN-Pbr clade-specific prophage homology group signature: a representative from the orthologous prophage group phage grp 107. (**d**) TPPbr clade-specific prophage homology group signatures comparison: two representative prophages from phage grp 46 group and regions from the five TP-Pbr genomes and the single FN-Pbr genome in which this prophage is absent are shown. For all figures, flanking genes are coloured white, and genes in the same homology groups are connected with vertical lines. Bacterial defence systems are marked as #, TA system, and ##, methyltransferase. BL diagnosis is marked as *, TP-Pbr, and **, FN-Pbr.

We further explored the prophages of isolates in the BL-causing clade, specifically to understand the intra-lineage variation and horizontal transfer with other *Pectobacterium* species. We detected four clusters with intact prophages exclusive to the BL-causing clade (Fig. 5a). First, a 49 kb intact prophage was found in all but two isolates from this clade (phage_grp_45 in Table S7). This prophage carried a YdaS/YdaT toxin–antitoxin (TA) system and a DNA methyltransferase gene, important elements of anti-phage defence systems (Fig. 5b). The same flanking genes are found across all genomes, which suggests a single insertion event of this prophage in the common ancestor of TP-Pbr and FN-Pbr isolates followed by vertical transmission.

A single FN-Pbr clade isolate sampled in 2019 in the Netherlands showed an insertion of a 50 kb prophage (phage_grp_343 in Table S7). Interestingly, this prophage had a very similar homology group signature to phage_grp_45 prophages, with some rearrangements, and was integrated at the same site in the host genome (Fig. 5b), implying a mosaic relationship. These prophages could potentially be clustered together when a relaxed threshold is applied. Nevertheless, they are distinct in gene content and structure, and our approach effectively captures phage mosaicism and dynamics across multiple genomes. Another 44 kb prophage was detected in all of the FN-Pbr isolates (phage_grp_107 in Table S7, Fig. 5c). In fragmented genome assemblies of seven FN-Pbr isolates, this prophage was split over two or more contigs. However, our pangenomic homology group signature matching allowed merging these fragments into a single prophage signature, demonstrating the use of a pangenomic approach in studying mobile genetic element dynamics. Interestingly, this prophage harboured a methyltransferase and antitoxin gene of the TA system, although the toxin component was missing.

Finally, a 31 kb prophage was detected exclusively in 30 TP-Pbr isolates collected between 2012 and 2020, mainly in the Netherlands (phage_grp_46 in Table S7. The five *P. brasiliense* isolates in this clade lacking this prophage were sampled in 2016 or later in Belarus and the Netherlands (Fig. 5a, d). Prophage signature comparison shows a remnant region in FN-Pbr genomes (g_406). Therefore, an insertion in the common ancestor of TP-Pbr and FN-Pbr clades and two independent excision events, first in the FN-Pbr clade and another in the five isolates of TP-Pbr clades, likely explains these results. In summary, we captured ongoing prophage and immune system dynamics in the clonal isolates based on the robust core phylogeny.

## Discussion

During the annual sampling of soft-rot *Pectobacteriaceae* in the Netherlands, we detected BL-causing *P*. *brasiliense* isolates that escaped the previously designed BL-phenotype assays [32]. To provide a broader context for the emergence of this isolate, we applied a pangenome approach to the *Pectobacterium* genus. Compared to the previous pangenome, we observed a stable core but an almost 50% increase in the accessory genome. Prophages contributed significantly to the *Pectobacterium* genus pangenome, mostly non-core genes. Further characterization of these prophages revealed the presence of both generalist and specialist prophages of *Pectobacterium* species.

The genetic diversity of *P. brasiliense* is evident from the broad range of intra-species ANI scores (94-99%). Pathogens can evade diagnosis by natural selection of isolates that acquire mutations in assay target genes. Diagnostic assay escape is not new for soft-rot pathogens. Previously, the Dia-A primer developed to detect *D. dianthicola* failed to detect a *Dickeya* outbreak in the USA because of target gene deletion [2]. In this and our case, the newly evolved isolates were very similar to those for which assays were developed (for *P. brasiliense*, 97.7% ANI). This highlights the challenges in designing stable phenotype diagnosis assays for soft-rot pathogens like *P. brasiliense* with high genomic diversity and an open pangenome. To address this, we propose monitoring as described in this study and periodic pangenome updates.

Bacterial species show a wide range in the proportion of genomes harbouring prophages, from 50% of the genomes in actinobacterial species harbouring one or more prophage-like elements [69] to 99.5% of *Acinetobacter baumanii* genomes [70]. The higher dissemination of prophages is known to drive the plasticity of bacterial genomes and contributes to pathogenicity and virulence [70]. In our collection, including *Pectobacterium* spp. isolates from all over the world, 99.7% of the genomes had one or more prophage-like elements, implying high genome plasticity at both genus and species levels. We further showed that prophages account for ∼16% of the *Pectobacterium* genus pangenome, one of the contributing factors to the growing pangenome. Bacteria and their phage predators are locked in an arms race, and it is possible that to counter these phages, prey bacteria are under selection pressure to acquire anti-phage defence systems harboured on mobile genetic elements such as plasmids, integrative conjugating elements and insertion sequences. We did not find convincing evidence for the presence of plasmids in *Pectobacterium* species, as we observed a discrepancy in plasmid prediction on high-quality versus fragmented genome assemblies. As prophages only explain ~16% of the pangenome, it remains to be seen whether other mobile genetic elements exist and how they interplay with the prophages in *Pectobacterium* species and contribute to pangenome growth.

We observed a significant difference in average genome size between the BL-causing and non-causing *P. brasiliense* isolates. Prophages explain only a quarter of this observed genome size difference. As observed in BL-causing lineages from our collection, lineage-specific prophages are also detected in other pathogens, for example, Φ*RS*551 in *Ralstonia solanacearum* [71] and Φ*RE*2010 in *S. enterica* [72]. Genes in such prophages are known to be transcriptionally active and up-regulated post-infection in different plant pathogens, for example, in *P. brasiliense* [73] and *R. solanacearum* [74,75]. These prophages modulate the mobility and virulence of bacterial pathogens and may provide a competitive advantage over other closely related species not bearing prophages [29,73]. Similar examples of horizontally acquired mobile elements modulating virulence and quorum sensing are also reported for the *Pectobacterium* species [29,76, 77]. Moreover, annual sampling across the Netherlands enabled us to capture the ongoing gain-loss dynamics of prophages, even within clonal lineages, and, therefore, highlights the contribution of prophages to genome plasticity. These lineage-specific prophages in *P. brasiliense* probably provide a competitive advantage over other closely related species, and the active dynamics of prophages enable such pathogens to continuously radiate novel lineages as observed.

We also showed the existence of generalist prophages targeting multiple *Pectobacterium* species. Recently, phages targeting multiple soft-rot *Pectobacterium* species were identified in Denmark [78]. Such prophage identification across species validates host recognition by the phage, an important characteristic for determining phage host range. Phage-host interaction networks often show modularity [68,79]. Taxonomic separation of hosts due to structural and metabolic differences is the intrinsic driver of such modular host ranges [64], emphasizing the need to include the host phylogeny while analysing phage host ranges. A pangenome approach to trace prophages shown here allows such paired study of host range and host phylogeny estimated from the core genome. These phage host range studies have important applications such as phage typing, controlling microbial community composition and phage therapy.

Biocontrol of plant pathogens using phages provides an environmentally friendly alternative to antibiotics [80]. Although lytic phages are more suitable phage therapy candidates, studying the dynamics of lysogenic phages will enable the learning of the host-detection mechanisms employed by phages in general. Recently, several studies have shown the effectiveness of this for *Pectobacterium* species and other plant pathogens [81,87]. However, just as with antibiotic resistance, the development of phage resistance in bacterial hosts [88] is a major bottleneck in phage therapy applications. Prophages also carry various defence systems, such as TA systems, restriction-modification systems and CRISPR [89,90], which can provide immunity to the host bacteria against both lytic and lysogenic phages [91]. All of the BL-causing *P. brasiliense* exclusive prophages harboured at least one defence system. Therefore, a comprehensive characterization of prophage diversity and its interplay with bacterial defence systems can pave the way for the smarter development of phage therapy applications. Additionally, a broader pangenome study that includes pathogens sharing niches with *Pectobacterium* species could help to discover prophages with cross-genus range.

However, such large-scale comparative analysis to study prophage dynamics comes with several challenges, such as reference bias and prophage overestimation because of fragmented assemblies [92]. Our approach overcomes these by combining prophage identification with the graph pangenome approach to merge fragmented prophages and correctly estimate their abundance in pangenome. We further used homology groups to identify orthologous prophages and constructed a non-redundant prophage set for *Pectobacterium* species. Thus, our approach provides a novel and efficient strategy to screen and select bacteriophage candidates, also for developing phage therapy applications.

In conclusion, our study demonstrates an important contribution of prophages in the growing *Pectobacterium* genus pangenome. We identified an ongoing bacteriophage-mediated exchange of genes in the new lineage of *P. brasiliense* as well as highly conserved prophage-like elements in the *P. brasiliense* genomes. Our results thereby emphasize the importance of routine sampling and pangenome analysis in understanding the evolutionary trajectories of bacterial plant pathogens.

## Supplementary material

10.1099/mgen.0.001392 Uncited Supplementary Material 1.

10.1099/mgen.0.001392 Uncited Supplementary Material 2.

## Abbreviations

ANI: average nucleotide identity
BL: blackleg
CTV: carotovoricin
CVP: crystal violet pectate
GO: gene ontology
PHROGs: Prokaryotic Virus Remote Homologous Groups
TA: toxin–antitoxin
