# Supplementary material for: Pangenomics to understand prophage dynamics in the Pectobacterium genus and the radiating lineages of Pectobacterium brasiliense
Source: Microb Genom. 2025 May 7;11(5):001392. doi: 10.1099/mgen.0.001392 (PMC12282264; doi:10.1099/mgen.0.001392)
Supplement: Uncited Supplementary Material 1. [file mgen-11-01392-s001.pdf]

Supplementary Figures, belonging to Pardeshi *et al.*, "Pangenomics to understand prophage dynamics in the *Pectobacterium* genus and the radiating lineages of *P. brasiliense*"

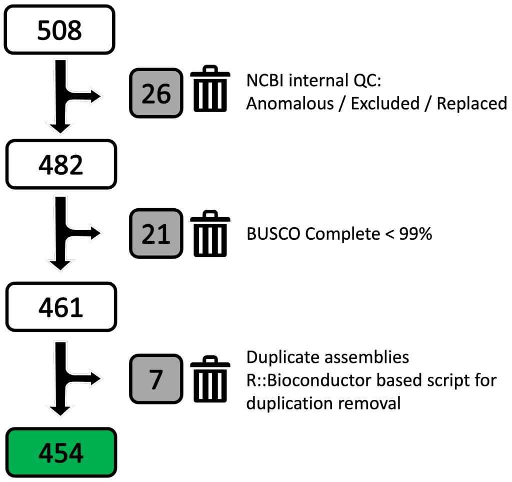

Supplementary Figure S1: Strategy used to filter genome assemblies during QC. Genome assemblies from NCBI that were marked as "anomalous", "excluded" or "replaced" were excluded. Incomplete genome assemblies with BUSCO completeness < 99% were excluded in second step. Finally, seven duplicate genome assemblies were removed using a custom R script.

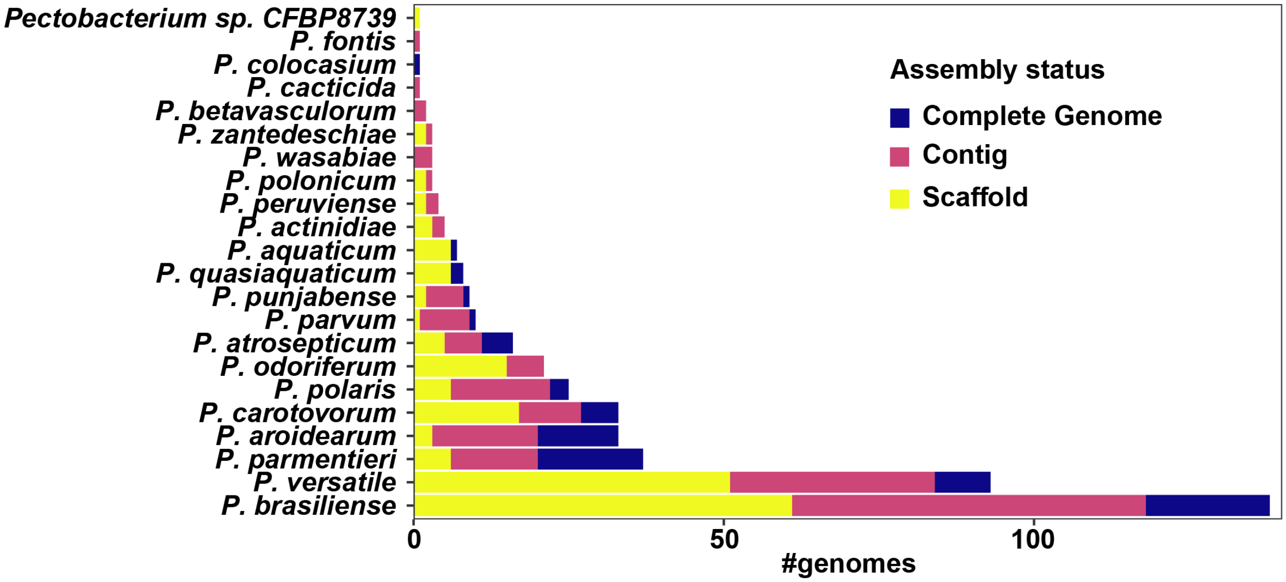

Supplementary Figure S2: Number of genome assemblies per *Pectobacterium* species in the pangenome.

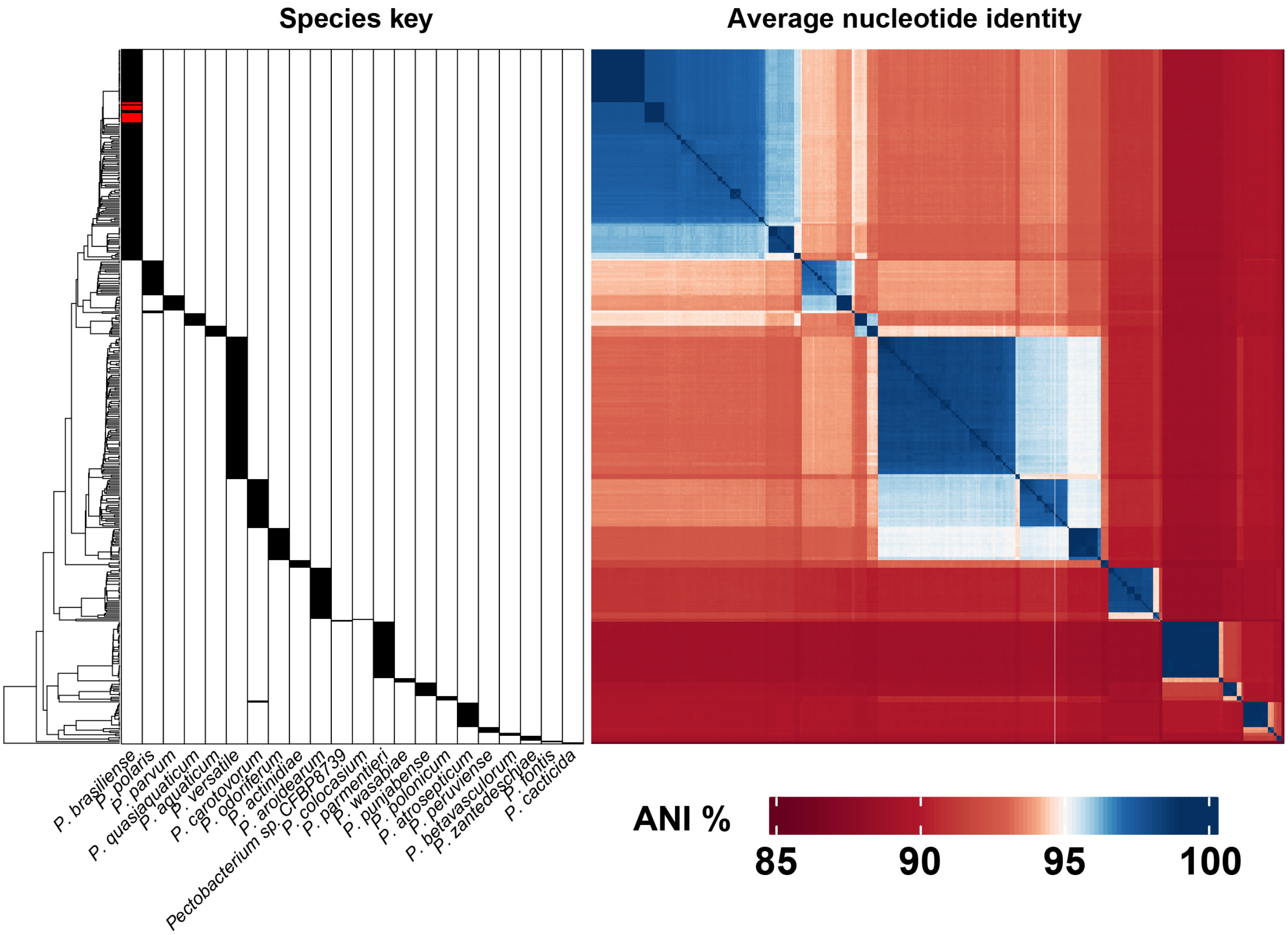

Supplementary Figure S3: ANI between all pairs of *Pectobacterium* spp. genomes. From left to right, a dendrogram based on UPGMA clustering on a (1-ANI) distance matrix; species key, where FN-Pbr isolates are marked in red; ANI heatmap. ANI heatmap color scale is centered around white color which represents 95% ANI, a commonly used species delineation cutoff.

Species	# genomes	Heaps' law alpha
P. brasiliense	138	0.538
P. polaris	25	0.646
P. versatile	93	0.501
P. carotovorum	33	0.577
P. odoriferum	21	0.727
P. aroidearum	29	0.751
P. parmentieri	37	0.823

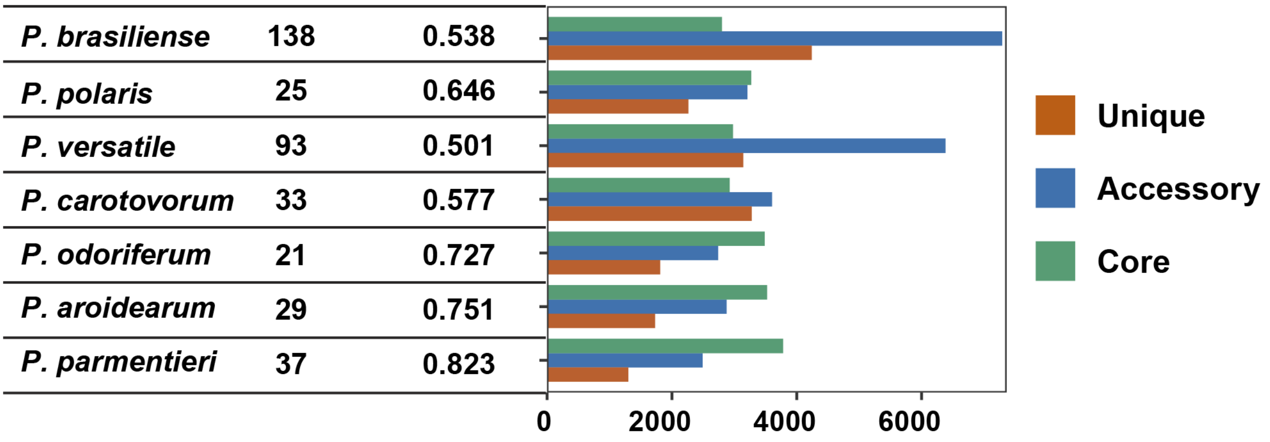

Supplementary Figure S4: Core, accessory and unique gene statistics for *Pectobacterium* species, with pangenome openness depicted by Heaps' law α value.

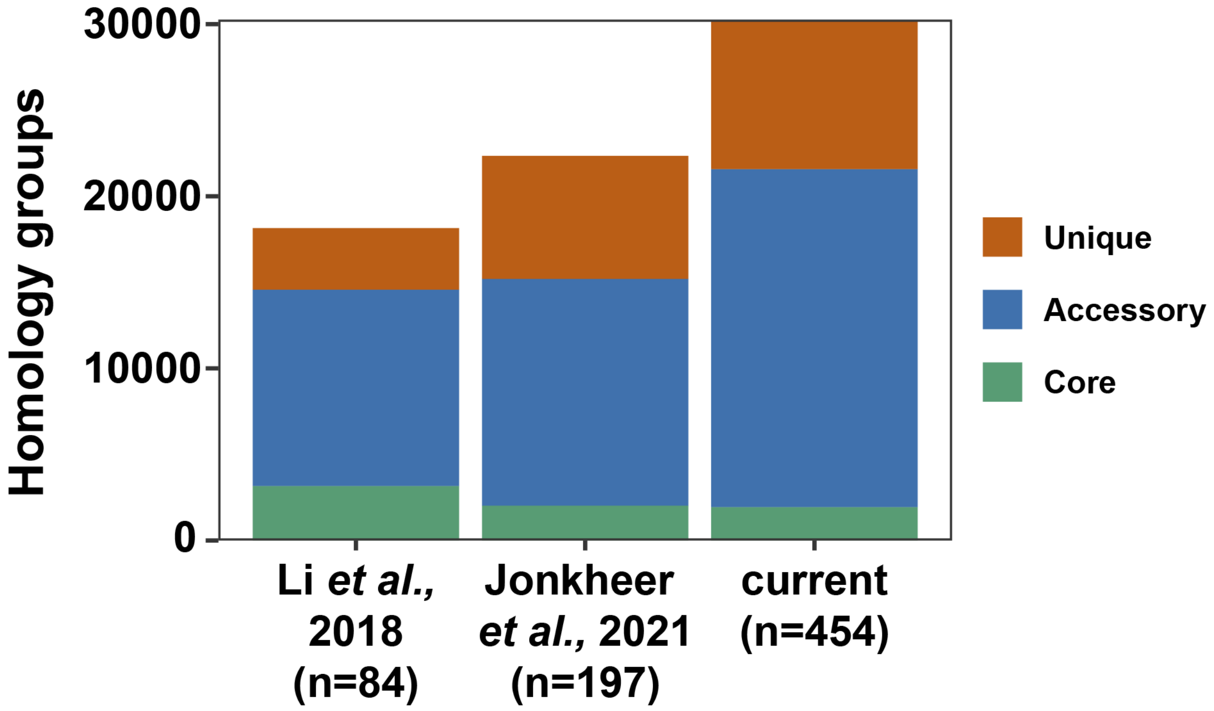

Supplementary Figure S5: Homology group counts in previous *Pectobacterium* genus pangenomes and the current one. The number of genomes in the pangenome are mentioned in parentheses.

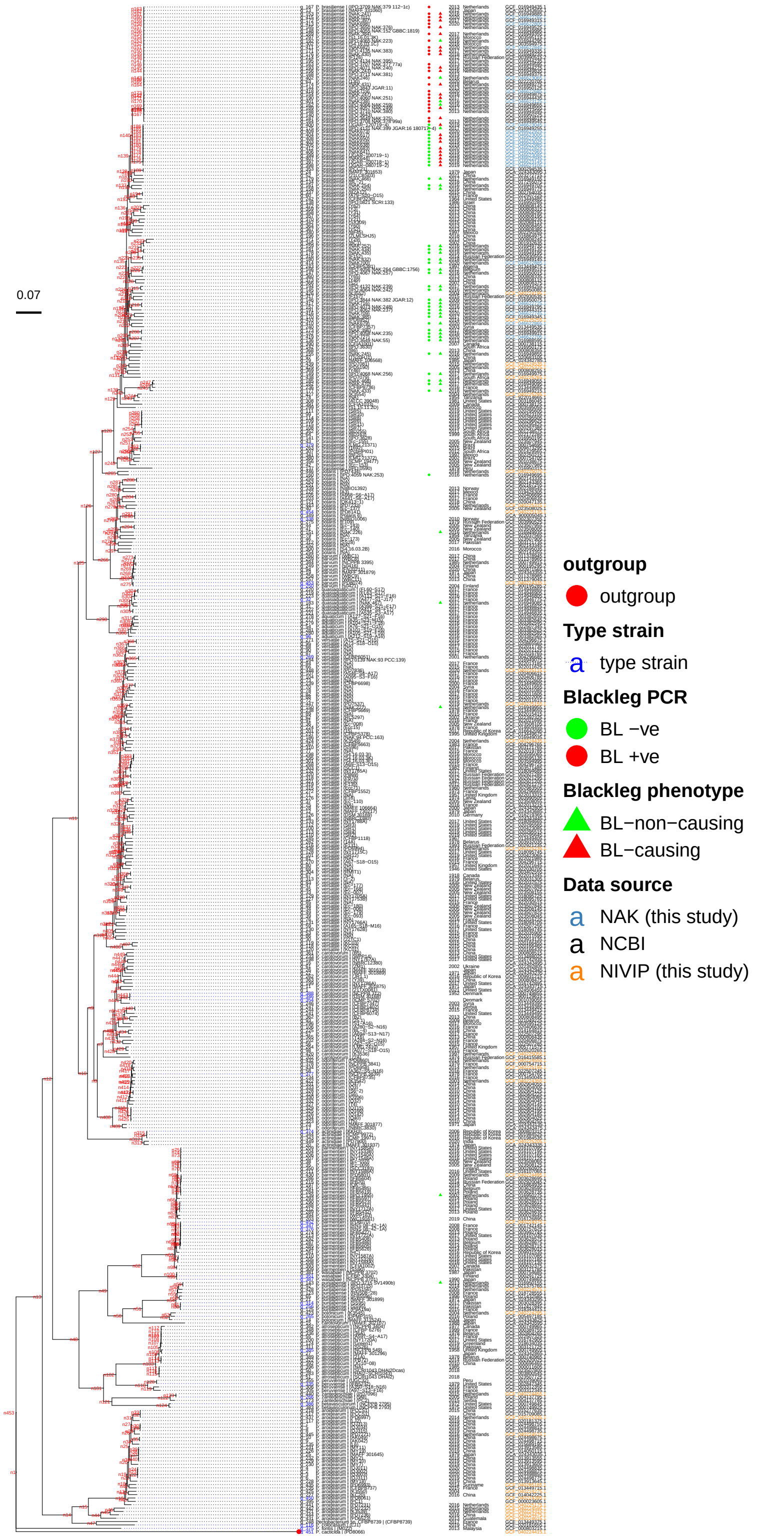

Supplementary Figure S6: Rooted core-SNP phylogenetic tree with annotated metadata. Internal nodes are labeled in red color and can be used for filtering Supplementary Table S1 using column 'nodepath.core_snp_ml'. Type strain leaf nodes are colored in blue. The leaf nodes show metadata from left to right: genome identifier, species name, strain name, BL-PCR, BL diagnosis during field trial, year of isolation of the strain, country of isolation and NCBI BioSample identifier. The BioSample identifiers are colored based on the source of data.

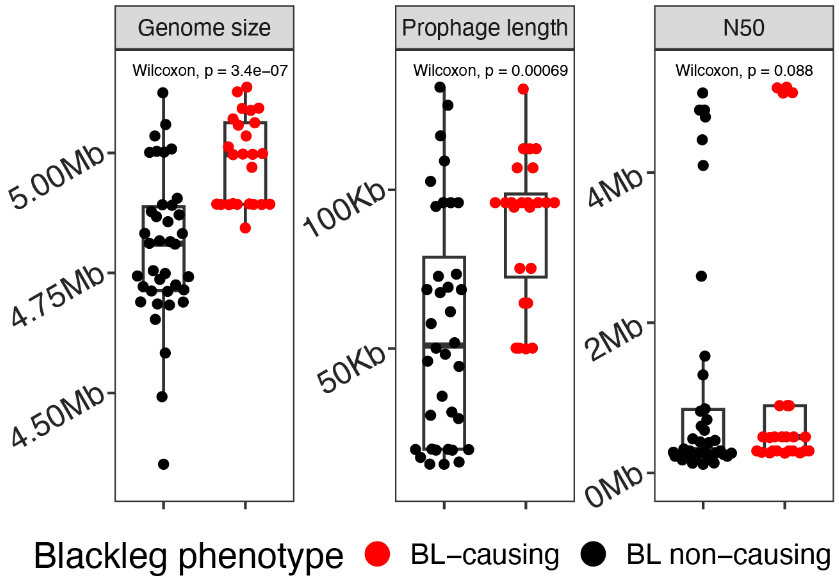

Supplementary Figure S7: Prophage length comparison between BL-causing and non-causing *P. brasiliense* isolates

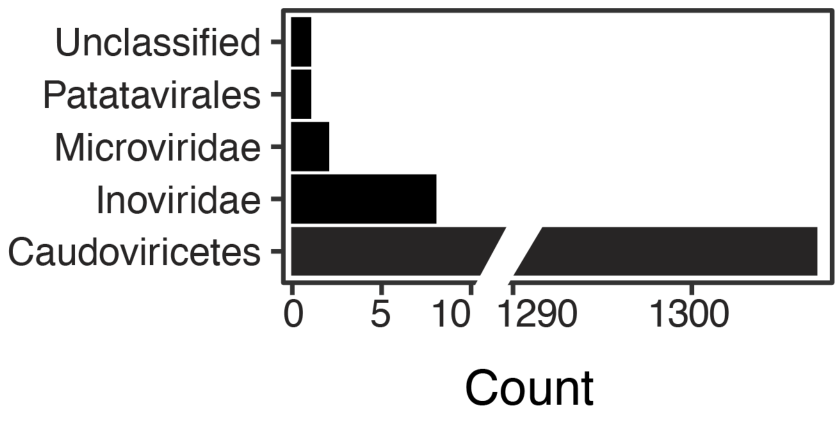

Supplementary Figure S8: Prophage taxonomy

(a) minChainLen = 5, maxGapLen = 2

```
seq1: a b c d e f g h i j k l m n o r s t
seq2: p q a b c d t f g h i l m n x y z i j k

LCS: a b c d f g h i l m n
Length: 11
Score: 47
Alignment:
a b c d – e f g h i j k l m n
a b c d t – f g h i – – l m n
```

(b) minChainLen = 5, maxGapLen = 2

```
seq1: a b c d e f g h i j k l m n o p q r s t
seq2: a b c d e f g h i x y z i j k l m

LCS: a b c d e f g h
Length: 8
Score: 40
Alignment:
a b c d e f g h
a b c d e f g h
```

(c) maxGapLen = 3

```
seq1: a b c d e f g h i j k l m n o p q r s t
seq2: m l k j z y x i h g f e d c b a

LCS: a b c d e f g h i j k l m
Length: 13
Score: 59
Alignment:
a b c d e f g h i – – – j k l m
a b c d e f g h i x y z j k l m
```

(d) minChainLen = 2

```
seq1: a b c d e f g h i j k l m n o p q r s t
seq2: m n

LCS: m n
Length: 2
Score: 10
Alignment:
m n
m n
```

Supplementary Figure S9: Syntenic Jaccard index calculation. To calculate the syntenic Jaccard index, first, the longest common subsequence (LCS) between two prophage homology group signatures is identified. Dynamic programing is used to calculate the LCS, where a score of +5 is used for a match and -2 for a mismatch of homology groups. The four examples (a-d) with dummy homology group signatures made of lower-case characters illustrate the impact of minimum chain length (minChainLen) and maximum gap length (maxGapLen) parameters on the LCS calculation. The number of matching homology groups in the final valid LCS is to calculate the syntenic Jaccard index.

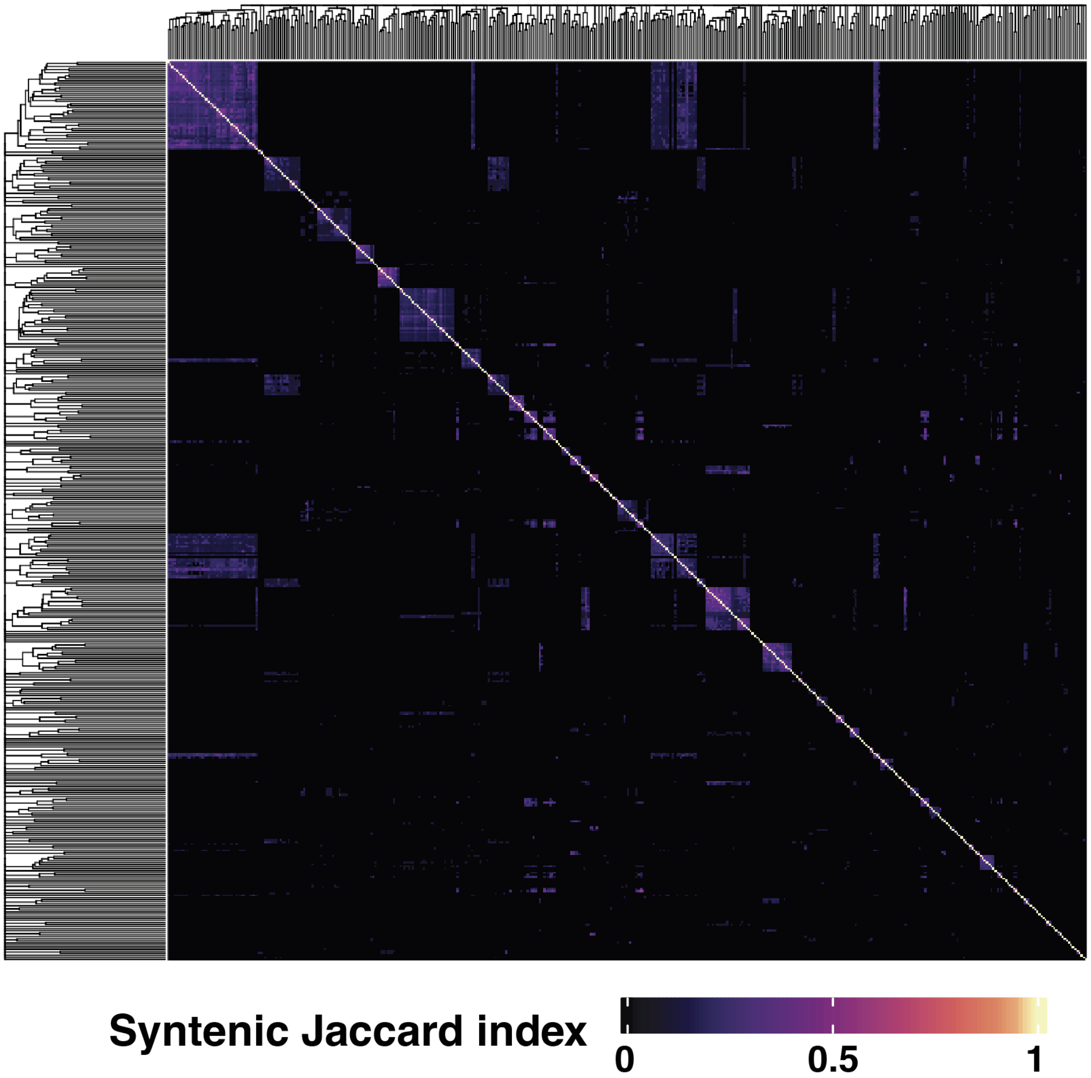

Supplementary Figure S10: Syntenic Jaccard index heatmap for 436 representative prophages.
